# Alpha-glucosidase inhibitors and hepatotoxicity in type 2 diabetes: a systematic review and meta-analysis

**DOI:** 10.1038/srep32649

**Published:** 2016-09-06

**Authors:** Longhao Zhang, Qiyan Chen, Ling Li, Joey S. W. Kwong, Pengli Jia, Pujing Zhao, Wen Wang, Xu Zhou, Mingming Zhang, Xin Sun

**Affiliations:** 1 Chinese Evidence-based Medicine Center, West China Hospital, Sichuan University, Chengdu 610041, China; 2 West China School of Public Health; Sichuan University, Chengdu 610041, China

## Abstract

Alpha-glucosidase inhibitors (AGIs) was reported to be associated with several rare adverse hepatic events, but with inconsistent results. We aimed to investigate the risk of hepatotoxicity associated with the use of AGIs in patients with type 2 diabetes mellitus (T2DM), and performed a systematic review and meta-analysis. Fourteen studies (n = 2881) were eligible, all of which were RCTs. Meta-analysis of data regarding elevation of more than 3-fold the upper limit of normal (ULN) of AST and ALT showed statistically significant differences between AGIs treatment versus control (OR 6.86, 95% CI 2.50 to 18.80; OR 6.48, 95% CI 2.40 to 17.49). Subgroup analyses of elevation of more than 1.8-fold ULN of AST and ALT by dose of AGIs showed differential effects on AST and ALT (AST: OR 0.38 vs 7.31, interaction P = 0.003; ALT: OR 0.32 vs 4.55, interaction p = 0.02). Meta-analysis showed that AGIs might increase the risk of hepatotoxicity, and higher dose appeared to be associated with higher risk of hepatotoxicity. However, the evidence is limited with surrogate measures (i.e. ALT and AST), and no clinically important adverse events were observed.

Alpha-glucosidase inhibitors (AGIs) are commonly used oral hypoglycemic drugs, especially in the patient population from East Asia^1^,^2^,^3^. The guideline of the American Diabetes Association (ADA) and the European Association for the Study of Diabetes (EASD) recommended the use of AGIs as a potentially first-line agent or in combination with other antihyperglycemic drugs^4^.

AGIs has proven similarly efficacious as other commonly used antidiabetes agents^5^,^6^,^7^. A recent large trial^1^ showed that acarbose is similar to metformin in terms of efficacy, and supports a viable choice for initial therapy in patients with newly diagnosed type 2 diabetes. Additionally, AGIs do not increase body weight, rarely cause hypoglycemia; and have minimal drug-drug interactions^1^,^7^,^8^.

Meanwhile, AGIs was reported to be associated with several rare adverse hepatic events^9^,^10^,^11^ and increase liver enzyme levels^12^,^13^,^14^,^15^,^16^,^17^,^18^. The causal relationship, however, has not been established^9^, and the magnitude of effect on the increase of liver enzyme levels remains unclear. Because these issues are often treated as adverse effects issues, and the hepatic adverse events, if any, are usually rare, individual trials are not adequate to address these important clinical questions. A meta-analysis – in which multiple studies are pooled – may offer opportunity to detect a small but clinically important difference.

Thus, we carried out a systematic review of randomized controlled trials and observational studies to assess the association between hepatotoxicity and AGIs. We hypothesized that hepatotoxicity would be more frequently manifested in AGIs as opposed to no use.

## Results

**Figure 1:**
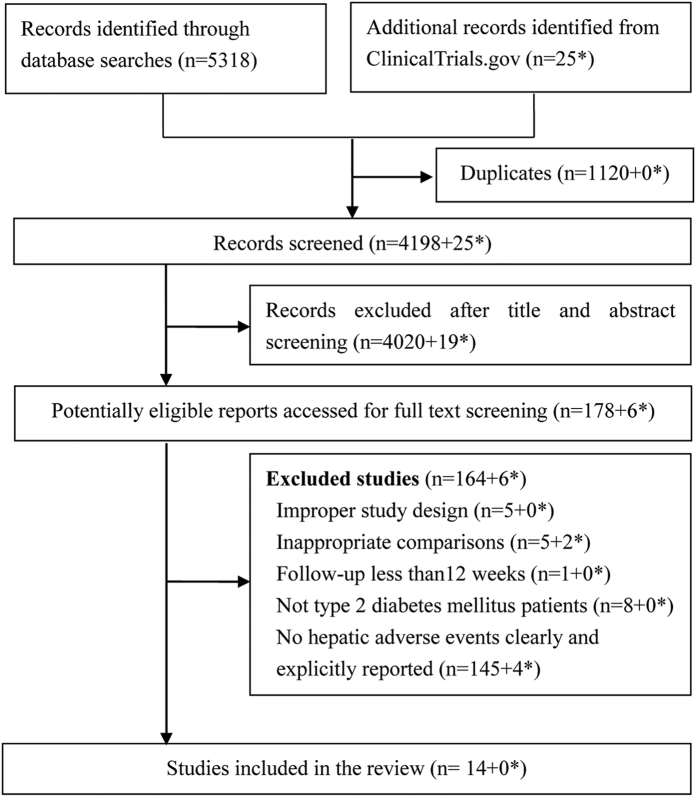
Flow chart of article selection. *Data form ClinicalTrial.gov.

Figure 1 showed the study selection process. We acquired 5,318 reports. After title and abstract screening, 178 were potentially eligible (including 159 potentially relevant RCTs and 19 potentially relevant observational studies^19^,^20^,^21^,^22^,^23^,^24^,^25^,^26^,^27^,^28^,^29^,^30^,^31^,^32^,^33^,^34^,^35^,^36^,^37^). Ultimately, 14 RCTs^13^,^14^,^15^,^16^,^17^,^18^,^38^,^39^,^40^,^41^,^42^,^43^,^44^,^45^ involving 2881 patients, proved eligible, and no observational studies were included.

### Study characteristics

Table 1 summarized the characteristics of included trials. Of those 14 RCTs, 10 (64.3%) were multicenter studies, and three (24.3%) were international trials. The length of follow-up ranged from 12 to 52 weeks. The trials enrolled 65 to 420 patients (total 3175), with a mean age range of 55.5 to 63.0, mean BMI 24.2 to 31.5 kg/m^2^, mean baseline HbA1c 6.5 to 9.6%, mean fasting plasma glucose 8.8 to 12.2 mmol/L, and mean duration of diabetes 1.8 to 12.2 years. Twelve tested acarbose, one tested miglitol and one tested voglibose. Twelve tested AGIS monotherapy, and two used AGIS as add-on or combination treatment.

### Risk of bias assessment

Table S1 (Appendix 2) summarized the risk of bias of included studies. The general reporting of methodological issues was suboptimal in those trials. Of those trials that provided adequate methodological details, two (14.2%) adequately generated random sequence; one (7.1%) adequately concealed allocation; 9(64.3%) blinded patients, caregivers, and outcome assessors. Incomplete rate of all patient ranged from 0 to 25.6%. The treatment groups of included trials were generally balanced with respect to demographics and clinical characteristics. Ten (71.4%) studies were industry funded.

### Risk of hepatotoxicity

Of the 14 RCTs reporting outcomes of interest, three^18^,^40^,^44^ explicitly stated no liver adverse events (AEs). Eleven studies reported changes of liver enzyme levels. We thus carried out meta-analyses regarding the high-grade (1.8-fold and 3-fold of ULN) AST and ALT elevation.

### High-grade AST elevation

**Figure 2:**
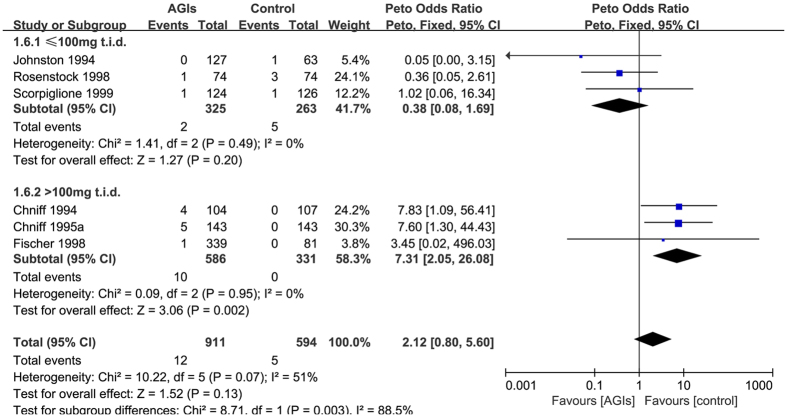
The subgroup analyses by the varying doses of AGIs on the elevations 1.8-fold the upper limit of normal of AST levels.

Six trials^13^,^15^,^39^,^43^,^44^,^45^ reported 17 cases elevations of more than 1.8-fold ULN of AST levels in 1505 patients who used at least one medication (raw event rate 1.0%). Pooling of these trials showed no statistically significant difference in the risk of elevations more than 1.8-fold ULN of AST levels between AGIs treatment and control (Peto OR: 2.12, 95% CI 0.80 to 5.60; I-square = 51%). The subgroup analyses by type of AGIs agent, type of control, length of follow-up and mode of treatment suggested no apparent differences, but differential effects were present among the varying doses of AGIs (AGIs ≤ 100 mg t.i.d. (Peto OR: 0.32, 95%CI 0.05 to 1.97) vs >100 mg t.i.d. (Peto OR: 7.31, 95%CI 2.05 to 26.08); interaction P = 0.003) (Fig. 2). The sensitivity analysis using alternative effect measures (OR vs RR), analysis models (random vs fixed) and pooling methods (Peto vs. Mantel-Hanszel method) did not show important changes in the pooled effects.

**Figure 3:**
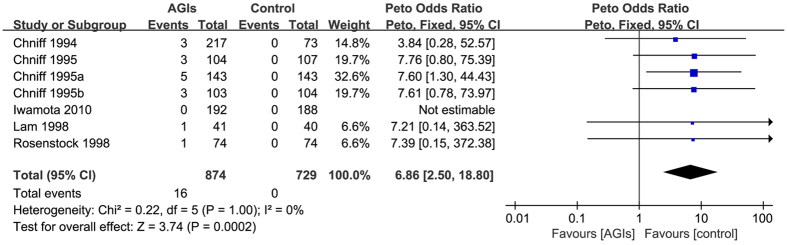
Alpha-glucosidase inhibitors on the elevations 3-fold the upper limit of normal of AST levels in patients with type 2 diabetes.

Seven trials^13^,^14^,^15^,^16^,^17^,^18^,^44^ reported 16 cases of elevation of more than 3-fold ULN of AST levels occurred in 1603 patients who used at least one medication (raw event rate 1.0%). Pooling of these trials showed an increased risk of elevations more than three times ULN of AST levels in patients taking AGIs versus control (Peto OR: 6.86, 95% CI 2.50 to 18.80; I-square = 0%) (Fig. 3). The subgroup analyses showed no any significant differences. The sensitivity analysis did not show important changes in pooled effects. In one of sensitivity analyses, we removed studies with potential overlap of study population across publications (e.g. Chniff 1995a and Chniff 1995b); the pooled estimates showed no significant change.

### High-grade ALT elevation

**Figure 4:**
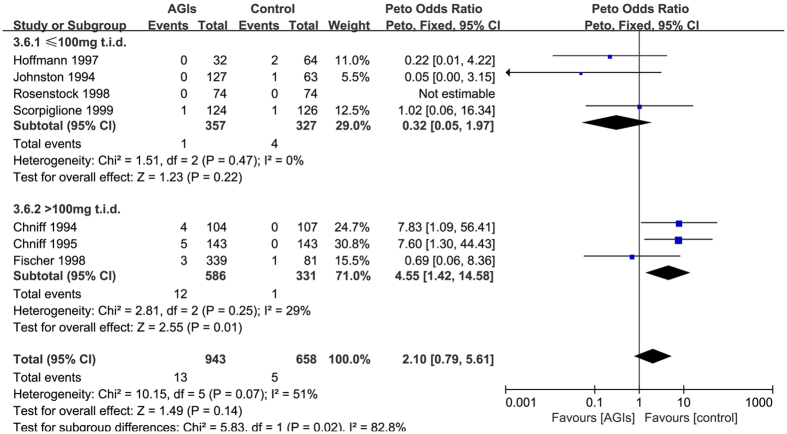
The subgroup analyses by the varying doses of AGIs on the elevations 1.8-fold the upper limit of normal of ALT levels.

Seven trials^13^,^14^,^39^,^41^,^43^,^44^,^45^ reported 18 cases elevations more than 1.8 times ULN of ALT levels occurred in 1601 patients who used at least one medication (raw event rate 1.1%). Pooling of these trials showed no statistically significant difference in the risk of elevations more than 1.8 times ULN of ALT levels between AGIs treatment and control (Peto OR: 2.10, 95% CI 0.79 to 5.61; I-square = 51%). The subgroup analysis by dose of AGIs ( AGIs ≤ 100 mg t.i.d. vs >100 mg t.i.d.) showed a relatively apparent differential effects (interaction P = 0.02, Peto OR: 0.32 (0.05 to 1.97) vs 4.55 (1.42 to 14.58) (Fig. 4). The sensitivity analysis did not show important changes in the pooled effects.

**Figure 5:**
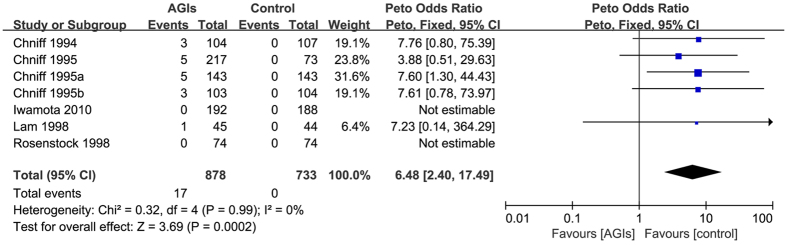
Alpha-glucosidase inhibitors on the elevations 3-fold the upper limit of normal of ALT levels in patients with type 2 diabetes.

Seven trials^13^,^14^,^15^,^16^,^17^,^18^,^44^ reported 17 cases elevations more than three times ULN of ALT levels occurred in 1611 patients who used at least one medication (raw event rate 1.0%). Pooling of these trials showed an increase in the risk of elevations more than three times ULN of ALT levels between AGIs treatment and control (Peto OR: 6.48, 95% CI 2.40 to 17.49; I-square = 0%) (Fig. 5). The subgroup analyses did not show any significant differences. The sensitivity analysis did not show important changes in the pooled effects, including the one analysis by removing studies with potential overlap of study population across publications (e.g. Chniff 1995a and Chniff 1995b).

## Discussion

In this study, we demonstrated the risk of hepatic AEs associated with AGIs use. Overall, the risk of developing hepatic AEs cannot be determined drawing from the 14 randomized trials, given the relatively small doses of acarbose ( ≤ 100 mg t.i.d), relatively small sample sizes and short follow-up. However, we found evidence showing increased risk of liver transaminases (AST and ALT) associated with the use of AGIs in patients with type 2 diabetes. In our study, there was a significant increase in odds of hepatotoxicity due to the elevation of AST and ALT. The odds of elevations greater than 3.0-fold ULN of AST and ALT levels were 6.86 and 6.48 times higher in patients receiving AGIs compared to patients in the control arms. For the pooling effects of elevations 1.8-fold ULN of AST and ALT, the results did not show a significant difference, but the subgroup analysis by dose of AGIs showed an apparent difference, which probably suggested that the risk of hepatotoxicity would be greatly increased when patients using AGIs more than 100 mg t.i.d. In addition, the study of Iwamoto^18^, which intervention was voglibose, showed no any changes in hepatic enzymes abnormalities ( ≥ 3-fold ULN range) of AST and ALT. In addition, some studies^4^,^13^,^14^,^15^,^16^,^17^ reported that these elevations were asymptomatic and became normalized after discontinuation of the study medication. Combined the result of subgroup analysis by dose of AGIs using, it probably indicated that there exists dose-response relationship between the hepatotoxicity and AGIs exposure dose.

Little research was available to explore the hepatotoxicity induced by AGIs. One review^7^ about hepatotoxicity have been published in 2007. As part of this review regarding safety profile of acarbose, the content just described that acarbose may lead to hepatotoxicity, while not performing a rigorous and thorough analysis to evaluate the relationship between acarbose and risk of hepatotoxicity. In our study, we also searched the FDA Adverse Event Reporting System^46^. The search yielded several cases of fatal hepatitis event in those using acarbose; however, additional information was unbailable. We also searched the US LiverTox website^47^, and found no any information regarding voglibose and absence of clinical acute liver injury in those using miglitol. The website reported a case of acute liver cell damage in a patient administered with acarbose. Nevertheless, all of above information was based on case reports, which was unable to offer causality.

Our study has a few strengthens. First, it offers an up-to-date and complete overview of randomized controlled trials and observational studies concerning AGIs treatment. Though we included a number of RCTs, the data are relatively consistent and heterogeneity is acceptable. In addition to published reports, we searched ClinicalTrials.gov for completeness of data^48^.In the meanwhile, one should interpret the findings cautiously because of limitations. First, many patients were withdrawal from these trials, possibly because of the side effect of gastrointestinal tract caused by AGIs, for which we were unable to account for^7^. Second, the reporting of hepatic AEs was lacking in many studies, leading to their exclusion from analysis. Adverse events, unlike efficacy outcomes, are rarely predetermined for systematic data collection in clinical trials. Therefore, reporting of adverse events depends highly on the investigators. Third, the type of reported hepatic AEs were highly variable, which made the collection and analysis of data challenging. Last, some studies^14^,^15^,^16^ have the potential overlap of study population across publications. We try to contact the author, but we can’t get the contact information. Meanwhile, we have carefully checked the studies for potential overlap of study population across publications, and found that the study designs of these articles differed with each other (including total number of patients, number of groups, tested group, control group, and usage of medication). We also conducted a sensitivity analysis by removing these studies with potential overlap participants; the pooled estimates showed no significant changes, suggesting robustness of results to this potential issue.

In conclusion, our meta-analysis suggested that patients taking acarbose could have higher risk of liver damage compared to patients without AGIs. Although not definitive, the findings may suggest caution in the use of AGIs for those who are at high risk for hepatic dysfunction. In summary, although the effects of AGIs on hepatic AEs remain uncertain, the randomized evidence consistently suggests increased risk of liver enzymes elevation in general. Dose-response relationship may exist between the hepatotoxicity and AGIs dose.

## Methods

### Eligibility criteria

We included randomized controlled trials (RCTs) and observational studies (cohort studies and case-control studies) of patients with type 2 diabetes mellitus without any liver disease or abnormal liver transaminase that compared alpha-glucosidase inhibitors (acarbose, miglitol, voglibose) with placebo, lifestyle modification, or active antidiabetic agents. An eligible study should also follow up patients for at least 12 weeks (not applicable to case-control studies), and explicitly report outcome data regarding any hepatic AEs (e.g. hepatitis, death, liver transplantation, hospitalization for hepatotoxicity or withdrawal due to any liver damage), or high-grade alanine transaminase (ALT) and aspartate transaminase (AST).

High-grade ALT and AST elevations were defined an elevation of more than 1.8-fold of the upper limit of normal (ULN)^13^,^14^,^15^,^16^,^39^,^42^,^49^,^50^. These liver transaminases (AST or ALT) are useful biomarkers of liver injury in a patient with some degree of intact liver function^50^,^51^,^52^.

### Literature search

We searched Medline, Embase, and the Cochrane Central Register of Controlled Trials (CENTRAL) for reports published in English language from inception to July 2015. We combined both Medical Subject Headings (MeSH) and free text terms for identifying relevant articles. An information expert (JJY) helped develop the search strategy (Appendix 1). In our search, we included search terms defining AGIs and T2DM only, as we had planned to evaluate all the potential adverse events of AGIs, including – but not limited to - hepatotoxicity. We also searched ClinicalTrials.gov for additional information, which provides important data on hepatotoxicity.

### Study process

Two reviewers, trained in health research methods, independently screened titles/abstracts and full texts for eligibility, assessed risk of bias, and collected data from each eligible study, using standardized, pilot-tested forms, together with detailed instructions. Reviewers resolved disagreement through discussion or, if required, adjudication by a third reviewer.

### Risk of bias assessment

We assessed the risk of bias of RCTs using the Cochrane Collaboration’s tool^53^. The items included random sequence generation, allocation concealment, blinding of participants, caregivers, and assessors of outcomes (i.e. hepatitis or changes in liver enzymes), incomplete outcome data, prognostic balance between treatment groups, selective reporting. We planned to assessed the risk of bias of observational studies using the Newcastle–Ottawa Quality Assessment Scale^54^.

### Data collection

We collected the following information from each study: study characteristics (authors’ name, year of publication, total number of patients randomization, number of treatment groups, length of follow-up, funding source, countries involved, and number of study sites); patient characteristics (gender, age, diabetes duration, body mass index (BMI), baseline HbA1c level, and fasting plasma glucose); interventions (medications common to all groups (baseline treatment, details of AGIs therapy and control group); and outcomes (any hepatic adverse events occurred during the course of study). For RCTs, if the initial treatment assignment was switched (e.g. patients in placebo group started receiving AGIs agents after 24 weeks), we collected the data prior to that point. If a trial had multiple reports, we collated all data into a single study^55^. If the outcome data were reported in multiple follow up points, we used data with the longest follow-up.

### Data analysis

We planned to analyze RCTs and observational studies separately. However, no observational studies were eligible. For randomized trials, we pooled the data using Peto’s methods because of the very low event rate^55^, and reported pooled Peto ORs and associated 95% CIs. We examined the heterogeneity among studies by the Cochran chi-square test and the I-squared statistic. We explored sources of heterogeneity with three priori subgroup hypotheses: type of AGIs agent (acarbose; miglitol and voglibose); type of control (AGIs vs placebo, AGIs vs active treatment); dose of AGIs using (AGIs ≤ 100 mg t.i.d. and >100 mg t.i.d.); length of follow-up ( ≤ 26 weeks and >26weeks) and mode of treatment (AGIs monotherapy, AGIs add-on/combination treatment). We carried out sensitivity analyses by using alternative effect measures (odds ratio (OR) vs. risk ratio (RR)), pooling methods (Peto vs. Mantel-Hanszel method), statistical models regarding heterogeneity (random vs. fixed effects), and removal of studies with potential overlap of study populations across publications.

We planned to examine publication bias by the funnel plot or other methods (such as Egger’s and Begg’s). Because of the low power of test associated with studies of low events rate, we were unable to examine this. We reported the results according to preferred reporting items for systematic reviews and meta-analyses (PRISMA)^56^.

## Additional Information

**How to cite this article**: Zhang, L. *et al*. Alpha-glucosidase inhibitors and hepatotoxicity in type 2 diabetes: a systematic review and meta-analysis. *Sci. Rep.* **6**, 32649; doi: 10.1038/srep32649 (2016).

## Supplementary Material

Supplementary Information

## Figures and Tables

**Table 1:** Characteristics of studies of AGIs treatment in patients with type 2 diabetes mellitus.

**Author(year)**	**International study**	**No of Groups**	**No of countries involved**	**No of study sites**	**Total No of patients**	**Follow up (weeks)**	**No (%) male**	**Mean age (years)**	**Mean BMI (kg/m^2^)**	**Mean HbA1c (%)**	**Mean FPG (mmol/L)**	**Mean diabetes duration (years)**	**Background medications**	**AGLS group**	**Control group**
Coniff (1994)^13^	NR	2	1	12	211	24	94(48.5)	55.80	31.5*	NR	11.50	0.5-33&	None	Acarbose 100 mg to 300mg t.i.d.	Placebo
Coniff (1995) 14	Yes	4	NR	NR	290	16	166(57.2)	55.49	30.50	8.96	12.09	5.50	None	Acarbose 100 mg to 300mg tid	Placebo
Coniff (1995a)^15^	NR	4	NR	NR	255	30	NR	55.90	29.86	6.92	12.18	5.37	None	Acarbose 200mg t.i.d.	placebo Tolbutamide
Coniff (1995b)^16^	NR	2	1	12	219	24	NR	NR	NR	6.51	9.65	NR	None	Acarbose 50mg to 300 mg t.i.d.	Placebo
Costa (1997)^38^	No	2	1	7	65	24	22(33.8)	60.87	28.12	8.91	10.27	NR	None	Acarbose 100 mg t.i.d.	Placebo
Fischer (1998)^39^	Yes	5	5	NR	420	24	222(52.9)	56.62	27.32	7.41^	NR	1.80	None	Acarbose 25mg to 200mg t.i.d.	Placebo
Gentile (2001)^40^	No	2	1	NR	100	26	NR	NR	27.8	8.80	8.78	9.0	None	Acarbose 100 mg t.i.d.	Placebo
Hoffmann (1997)^41^	No	3	1	4	96	24	30(31.2)	58.33	26.70	9.57	8.87	2.92	None	Acarbose 100 mg t.i.d.	Metformin
Hwu (2003)^42^	Yes	2	2	6	111	18	55(49.5)	56.32	24.15	9.50	10.54	12.21	None	Acarbose 100mg t.i.d	Placebo
Iwamota (2010)^18^	No	2	1	51	380	12	251(66.1)	59.14	24.54	7.55	9.00	5.35	None	Voglibose 0.2mg t.i.d	Vildagliptin
Johnston (1994)^43^	No	3	1	12	192	20	109(56.8)	58.33	30.32	8.85	11.04	8.64	None	Miglitol 50 mg to 100mg t.i.d.	Placebo
Lam (1998)^17^	No	2	1	3	89	24	39(43.8)	57.36	24.45	9.45	10.46	10.15	None	Acarbose 100mg t.i.d.	Placebo
Rosenstock (1998)^44^	NR	2	1	NR	168	24	78(52.7)	56.55	29.70	8.32	9.76	7.50	Metformin	Acarbose 50 mg to 100 mg t.i.d.	Placebo
Scorpiglione (1999)^45^	No	2	1	17	250	52	123(49.2)	62.99	31.5	8.55	11.47	10.45	Standard care	Acarbose 100 mg t.i.d.	No additonal drugs

* = Median; & = full range; ^ = Geometric mean; NR = not report.
