# Supplementary material for: Alpha-glucosidase inhibitors and hepatotoxicity in type 2 diabetes: a systematic review and meta-analysis
Source: Sci Rep. 2016 Sep 6;6:32649. doi: 10.1038/srep32649 (PMC5011653; doi:10.1038/srep32649)
Supplement: Supplementary Information [file srep32649-s1.doc]

**Alpha-glucosidase inhibitors and** **hepatotoxicity in** **type 2 diabetes: a systematic review and meta-analysis**

Authors

Longhao Zhang^1^, Qiyan Chen^2^, Ling Li^1^, Joey S.W. Kwong^1^, Pengli Jia^1^, Pujing Zhao^1^, Wen Wang^1^, Xu Zhou^1^, Mingming Zhang*^1^, Xin Sun*^1^

Affiliations

1. Chinese Evidence-based Medicine Center, West China Hospital, Sichuan University, Chengdu 610041, China
2. West China School of Public Health; Sichuan University; Chengdu 610041; China

Correspondence to:

1. X Sun, Chinese Evidence-Based Medicine Center, West China Hospital, Sichuan University, 37 Guo Xue Xiang, Chengdu 610041, Sichuan, China

Email: sunx79@hotmail.com

1. MM Zhang, Chinese Evidence-Based Medicine Center, West China Hospital, Sichuan University, 37 Guo Xue Xiang, Chengdu 610041, Sichuan, China

Email: mingming-zhang@163.com

**Supplementary information**

**Appendix 1** Search strategies

**Appendix 2** Table S1

**Appendix 1 Search strategies**

1. **MEDLINE (Ovid)** **(**Search date: July, 2015**)**
2. acarbose/ or acarbose.mp.
3. miglitol.af
4. voglibos*.af.
5. alpha-glucosidase inhibitor.af.
6. glucosidase inhibitors.af.
7. bayg5421.af.
8. bay5421.af.
9. glucobay.af.
10. precos*.af.
11. prandas*.af.
12. akarbos*.af.
13. glyset.af.
14. baym1099.af.
15. bay1099.af.
16. emiglitat*.af.
17. basen.af.
18. sk-983.af.
19. bay-m-1099.af.
20. plumarol.af.
21. 1 or 2 or 3 or 4 or 5 or 6 or 7 or 8 or 9 or 10 or 11 or 12 or 13 or 14 or 15 or 16 or 17 or 18 or 19
22. diabetes mellitus, type 2/
23. diabetes mellitus, type 2.tw.
24. type 2 diabetes mellitus.tw.
25. niddm*
26. mody*
27. dmt2
28. t2dm
29. stable diabet*
30. 21 or 22 or 23 or 24 or 25 or 26 or 27 or 28
31. 20 and 29
32. **embase (Ovid) (**search date: July 25, 2015**)**
33. acarbose.mp. or acarbose/
34. miglitol.tw.
35. voglibos*.tw.
36. alpha-glucosidase inhibitor.tw.
37. glucosidase inhibitors.tw.
38. bayg5421.tw.
39. bay5421.tw.
40. glucobay.tw.
41. precos*.tw.
42. prandas*.tw.
43. akarbos*.tw.
44. glyset.tw.
45. baym1099.tw.
46. bay1099.tw.
47. emiglitat*.tw.
48. basen.tw.
49. sk-983.tw.
50. bay-m-1099.tw.
51. plumarol.tw.
52. 1 or 2 or 3 or 4 or 5 or 6 or 7 or 8 or 9 or 10 or 11 or 12 or 13 or 14 or 15 or 16 or 17 or 18 or 19
53. non insulin dependent diabetes mellitus/
54. diabetes mellitus, type 2.tw.
55. type 2 diabetes mellitus.tw.
56. niddm*tw.
57. mody*.tw.
58. dmt2.tw.
59. t2dm.tw.
60. stable diabete*.tw.
61. 21 or 22 or 23 or 24 or 25 or 26 or 27 or 28
62. 20 and 29
63. **cochrane central register of controlled trials** **(Ovid)** July 2015)
64. acarbose/ or acarbose.mp.
65. miglitol.af
66. voglibos*.af.
67. alpha-glucosidase inhibitor.af.
68. glucosidase inhibitors.af.
69. bayg5421.af.
70. bay5421.af.
71. glucobay.af.
72. precos*.af.
73. prandas*.af.
74. akarbos*.af.
75. glyset.af.
76. baym1099.af.
77. bay1099.af.
78. emiglitat*.af.
79. basen.af.
80. sk-983.af.
81. bay-m-1099.af.
82. plumarol.af.
83. 1 or 2 or 3 or 4 or 5 or 6 or 7 or 8 or 9 or 10 or 11 or 12 or 13 or 14 or 15 or 16 or 17 or 18 or 19
84. diabetes mellitus, type 2/
85. diabetes mellitus, type 2.tw.
86. type 2 diabetes mellitus.tw.
87. niddm*
88. mody*
89. dmt2
90. t2dm
91. stable diabet*
92. 21 or 22 or 23 or 24 or 25 or 26 or 27 or 28
93. 20 and 29

**ClinicalTrials.gov** (Search date: October, 2015)

Completed | Studies With Results | acarbose OR bayg5421 OR glucobay OR precose OR miglitol OR glyset OR baym1099 OR voglibose OR alpha-glucosidase inhibitor OR glucosidase inhibitors OR basen OR SK-983 OR Bay-m-1099 OR plumarol

**Appendix 2**

| **Table S1 Risk of bias of randomized controlled trials of AGIs treatment and hepatotoxicity in patients with type 2 diabetes mellitus** | | | | | | | | | |
| --- | --- | --- | --- | --- | --- | --- | --- | --- | --- |
| **Author(year)** | **Randomization**  **sequence generation** | **Allocation concealment** | **Blinding** | | **Incomplete outcome data** | | **Incomplete rate of all patients (incomplete NO./total NO.)** | **Baseline comparability** | **Types of sponsors** |
|  |  |  | **Patients and care providers** | **outcome assessors** | **AGLS group** | **Comparator group** |  |  |  |
| Coniff(1994) ^13^ | Unclear risk | Unclear risk | Low risk | Low risk | 27/104; | 27/107; | 25.6%(54/211) | Comparable | Private for-profit funding |
| Chniff(1995)^14^ | Unclear risk | Unclear risk | Low risk | Low risk | Unclear | Unclear | Unclear | Comparable | NR |
| Coniff(1995)a^15^ | Unclear risk | Unclear risk | Low risk | Low risk | Unclear | Unclear | 12.1%(35/290) | Comparable | Private for-profit funding |
| Coniff(1995)b^16^ | Unclear risk | Unclear risk | Unclear risk | Unclear risk | Unclear | Unclear | 5.5%(12/219) | Comparable | Private for-profit funding |
| Costa(1997) ^38^ | Unclear risk | Unclear risk | Low risk | Low risk | 12/36 | 2/29 | 21.5%(14/65) | Comparable | Private for-profit funding |
| Fischer(1998) ^39^ | Unclear risk | Unclear risk | Low risk | Low risk | 32/339 | 11/81 | 8.7%(43/495) | Comparable | NR |
| Gentile(2001) ^40^ | Unclear risk | Unclear risk | Low risk | Low risk | 0/52 | 0/48 | 0% | Comparable | Public funding |
| Hoffmann(1997)^41^ | Low risk | Unclear risk | Low risk | Low risk | 3/31 | 2/63 | 5.2%(5/96) | Comparable | NR |
| Hwu(2003) ^42^ | Unclear risk | Unclear risk | Unclear risk | Unclear risk | Unclear | Unclear | 8.9%(10/112) | Comparable | Private for-profit funding |
| Iwamota(2010)^18^ | Unclear risk | Unclear risk | Low risk | Low risk | 9/192 | 10/188 | 5%(19/380) | Partial comparable | Private for-profit funding |
| Johnston(1994) ^43^ | Unclear risk | Unclear risk | Unclear risk | Unclear risk | 13/129 | 0/63 | 6.8%(13/192) | Comparable | Private for-profit funding |
| Lam(1998) ^17^ | Unclear risk | Unclear risk | Low risk | Low risk | 5/45 | 5/44 | 11.1%(10/90) | Comparable | Private for-profit funding |
| Rosenstock(1998)^44^ | Unclear risk | Unclear risk | Unclear risk | High risk | 10/74 | 10/74 | 11.9%(20/168) | Comparable | Private for-profit funding |
| Scorpiglione(1999)^45^ | Low risk | Low risk | High risk | High risk | 13/124; | 17/126 | 14%(35/250) | Partial comparable | Public funding and Private non-profit funding |
